# Hypertensive Disorders in Pregnant Women Receiving Fertility Treatments

**DOI:** 10.22074/ijfs.2018.5232

**Published:** 2018-03-18

**Authors:** Maryam Barekat, Shahnaz Ahmadi

**Affiliations:** 1 Department of Cardiovascular, Bushehr University of Medical Sciences, Bushehr, Iran; 2 Department of Regenerative Biomedicine, Cell Science Research Center, Royan Institute for Stem Cell Biology and Technology, ACECR, Tehran, Iran; 3 Department of Obstetrics and Gynecology, Iran University of Medical Sciences, Tehran, Iran

**Keywords:** Gestational Hypertension, Hypertension, Infertility, Preeclampsia, Pregnancy

## Abstract

Hypertensive disorders (HDs) as the most prevalent medical problem during pregnancy, predispose the patient to a lot of comorbidities and may even cause maternal or fetal death. The rate of infertility has been increasing in recent decades. So, we collected and summarized data about the co-existence of these two entities and found that HDs are somewhat more common in women receiving fertility treatments regardless of pathophysiologic correlation of infer- tility and hypertension or older age and chance of multiple pregnancies.

## Introduction

Hypertensive disorders (HDs) are the most prevalent medical problem during pregnancy. It is estimated that HDs involve up to 6-8% of all pregnancies (1). HDs account for about 25% of all pre-birth hospital admissions (2). It has been well-documented that hypertension plays an important role in development of atherosclerosis consequently leading to nonfatal or fatal myocardial infarction and cerebrovascular accidents. It has also been shown that hypertension is the main cause of perinatal and maternal morbidity and mortality including intrauterine growth retardation (IUGR), Hemolysis, Elevated Liver enzymes, and Low Platelet count (HELLP) Syndrome, renal impairment, premature labor, neonatal intensive-care-unit admission, caesarean section, placental abruption, perinatal death and maternal convulsion (3-5).

In a recent retrospective study done in Ethiopia, Seyom et al. (6) reported rate of dead fetus, low birth weight and low APGAR score, abortion, preterm delivery and HELLP syndrome as 10.2, 30.5, 18.5, 10.7, 31.4 and 12.4%, respectively in 55,860 pregnant women with HDs.

Zibaeenezhad et al. (7) found a prevalence of 2.32% for HDs in pregnant women in south of Iran including a prevalence of 2.13% for chronic hypertension. Moreover, Khosravi et al. (8) reported a prevalence of 9.8% for HDs among pregnant women who were admitted to a tertiary center in Tehran for delivery. So, the disease is also prevalent in Iran.

Infertility is also a common condition and physicians are deeply concerned about it, because it involves a couple, rather than a single individual. It is defined as inability of a couple to conceive after one year of regular intercourse without using any form of contraception (9).

The prevalence of infertility is markedly high in Eastern Europe, North Africa, Oceania and sub Saharan Africa (10). The main causes of infertility include male factors, decreased ovarian reserve, ovulatory factors, tubal factors, uterine factors, pelvic factors, and unexplained reasons (11).

Once the pathologic basis of infertility is recognized, therapy is directed toward curing reversible causes and modifying irreversible etiologies. Therapeutic interventions for both male and female infertility includes drug therapy (12) and surgery (13), with or without procedures like intra uterine insemination (IUI) or in vitro fertilization (IVF) (14, 15). 

### Methodology

We searched PubMed and Google search engines for incidence of hypertension and also history of infertility in pregnant women. We also checked the internet for causes of female infertility and their association with hypertension, all kinds of treatments and medications that are applied for female infertility and the chance and the mechanisms by which they changing blood pressure (BP). Then we quested for general considerations, treatment modalities and follow-up in pregnant cases with HDs, with or without a history of infertility.

Physiological blood pressure changes during pregnancy Normotensive women usually experience about 5 to 10 mmHg fall in their BP starting from the first trimester which may be continued up to the third trimester; after that, BP is restored to its preconception level (16). This is due to marked vasodilation which can overcome the increment of blood volume in this period. This phenomenon can also induce normal BP in cases with mild chronic hypertension which results in reduction in dose or discontinuation of antihypertensive medications or even masking previously undiagnosed cases.

### Diagnosis and risk of hypertension

Hypertension is generally labeled when systolic BP is ≥140 mmHg and/or diastolic BP is ≥90 mmHg, according to the mean of at least two measurements, checked using the same arm with at least fifteen minutes intervals, in clinic or in hospital (17). Although the definition of HDs is somewhat different in some references and defined only when diastolic BP is greater than 90 mmHg on two sessions with more than 4 hours interval or when a single diastolic BP >110 mmHg was recorded (18). BP should be measured in the sitting position while the arm is at the level of the heart, using a cuff of appropriate size. Mild hypertension is defined as a diastolic BP of 90-99 mmHg and/ or a systolic BP of 140-149 mmHg. Severe hypertension is defined as a systolic BP of ≥160 mmHg or a diastolic BP of ≥110 mmHg. Obviously, moderate hypertension ranges between mild and moderate values (Table 1) (17, 19, 20).

**Table 1:** Grading of severity of hypertension and the need for antihypertensive treatment


Grade of hypertension	Blood pressure levels (mm Hg)	Treat	Grade of treatment

Mild	Diastolic: 90-99	No^*^	Not applicable^*^
	Systolic: 140-149		
Moderate	Diastolic: 100-109	Yes	<150 systolic^*^
	Systolic: 150-159		<100 diastolic^*^
Severe hypertension	Diastolic: ≥110	Yes	<150 systolic^*^
	Systolic: ≥160		<100 diastolic^*^


*; Except for women with chronic hypertension with end-organ damage who should be treated even if blood pressure is mild and the goal is to normalizing their blood pressure. Modified from: hypertension in pregnancy: the NICE guidelines (20).

HDs are classified into four major groups according to working group of National Institutes of Health (NIH) report on high BP in pregnancy (3): i. Chronic or pre- existing hypertension diagnosed either before pregnancy or earlier than 20 gestational weeks, ii. Preeclampsia- eclampsia. Preeclampsia described as the presence of hypertension, along with new-onset of significant proteinuria of >0.3 g/24 hours. However, there are some other definitions in other references which are more precise and complete, iii. Preeclampsia superimposed on chronic hypertension, and iv. Gestational hypertension is defined as a hypertension beginning at later than 20 gestational weeks and can persist for up to 42 days post- partum.

It has also been mentioned that gestational hypertension usually resolves within up to 12 weeks post-partum however this is not applicable for cases with chronic hypertension (21).

Risk factors for chronic hypertension are: early middle age or about age 45, black race, using tobacco, too much salt (sodium) in diet, too little potassium, calcium or vitamin D in diet, drinking too much alcohol, high levels of stress, being overweight or obese, little or no exercise, history of high BP in the family (22). Certain chronic conditions also may increase the risk of high BP, such as kidney disease, diabetes and sleep apnea.

Risk factors for preeclampsia include: prior history of preeclampsia, family history of preeclampsia, intervals of more than 10 years between pregnancies, nulliparity, pre- existing medical conditions like antiphospholipid syndrome, type 1 or 2 diabetes mellitus, chronic kidney disease, chronic hypertension, chronic autoimmune diseases like systemic lupus erythematous (SLE), mother age more than 40 years, BMI >35 kg/m2, multiple pregnancy, high BP in the first visit, gestational trophoblastic disease, fetal triploidy (23, 24).

Thus, infertility by itself is neither a major risk factor for HDs during pregnancy nor a major risk factor for preeclampsia.

### Hypertension and infertility

Higher rates of HDs in women who underwent infertility treatment might be due to higher age and/or increased risk of multiple pregnancies. Also, pathologic basis of infertility like polycystic ovaries and endometriosis, must be considered as a cause of or in correlation with hypertension. In these conditions, hypertension may simply occur due to associated obesity or insulin resistance, androgen excess, sympathetic nerve over activity and chronic use of oral contraceptives (25, 26).

Furthermore, chronic hypertension can cause poor egg quality; also, many hypertensive women suffer from obesity which is mostly a result of excessive estrogen production which can lead to infertility. Antihypertensive medications like angiotensin receptor inhibitors (ARBs) and calcium channel blockers typically affect male fertility rather than female ones.

The most common medications which are used for treatment of female infertility are clomiphene, metformin, aromatase inhibitors like letrozole, human chorionic gonadotropins (hCG) like menotropin, dopamine agonists like bromocriptine and gonadotropin-releasing hormone (GnRH) agonists like leuprolide which is used in GnRH protocol and consists of progesterone and estradiol. Among these, letrozole, leuprolide and estradiol can induce hypertension with a prevalence rate of 5-8% (27-29), 8% (30) and 3-7% (31), respectively. Although bromocriptine usually causes vasodilatation and specially edema thereafter, there are some case reports on bromocriptine- induced hypertension (32). 

It is well known that estrogen-containing medications can induce hypertension in premenopausal women, but the mechanisms are not fully understood. Supraphysiologic concentrations of estrogen and its effect on increment of angiotensinogen and insulin-like growth factor I production by liver, increased sympathetic activity and increased expression of angiotensin subtype 1 (AT1) receptor in the kidneys, are the possible mechanisms (33).

In recently published meta-analysis, it was shown that metformin decreases BP specially systolic type, particularly in nondiabetic cases (34).

Farland et al. (35) reported relative risk of hypertension in infertile women receiving different kinds of treatments, as follows: clomiphene: relative risk (RR)=0.97, confidence interval (CI): 0.90-1.04; gonadotropin alone: RR=0.97, CI: 0.87-1.08, IUI: RR=0.86, CI: 0.71-1.03, IVF: RR=0.86, CI: 0.73-1.01. 

We could not find any correlation between risk of hypertension and hCG administration after adjustment by higher chance of multiple pregnancy and other prevalent factors.

In a meta- analytic study which was done in Germany, oocyte donation was also reported as a risk factor for HDs in pregnancy and this effect was possibly mediated through immunological processes and ovarian dysfunction (36).

In 1994, Sealey et al. (37) revealed that renin and urinary aldosterone excretion had 5-fold increases during the luteal phase (day 7) in patients who underwent ovarian stimulation. Alternatively, Tollan et al. (38) showed a statistically significant decrement in both systolic and diastolic BP during ovarian stimulation for IVF, most probably due to decreased level of adrenalin.

In a retrospective observational cohort, Hernández-Díaz et al. (39) interviewed 5151 women within six months of delivery and stated that the incidence of gestational hypertension was significantly greater in women with a history of infertility who were treated for this problem (15.8%) than among those infertile cases who did not receive such managements (8.9%). Results were the same for patients with preeclampsia and also after adjustment for age, twin pregnancy, parity and body mass index. There were some cases without a history of infertility treatments who mentioned some difficulties in fertility in past or untreated sub-fertility in the present pregnancy. Surprisingly, these women were not at increased risk of hypertension and this finding demonstrates the direct role of infertility treatment in induction of hypertension. All kinds of infertility treatment approaches or drugs were associated with a similar increased risk, although not unexpectedly, treatments with the greatest chance of multiple gestations, were associated with higher risk of HDs.

Alternatively, in a prospective cohort study, Farland et al. (35) included 116,430 women and followed them for 20 years to assess the risk of development of hypertension in later life. Among them, 12,183 received some kinds of infertility treatment. During follow-up, approximately 20,066 women were diagnosed with hypertension. The authors emphasized that only infertility due to tubal causes was accompanied by greater risk of hypertension as these cases had 15% greater risk of hypertension than women without a history of infertility. But among other causes of infertility, no clear relation was detected between receiving fertility treatment and subsequent hypertension. 

Meanwhile, Toshimitsu et al. (40) showed that the incidence of HDs was significantly higher in infertile couples with IVF/intracytoplasmic sperm injection conception than the spontaneous conception group in both the women aged ≥40 years (20.5 vs. 7.9%) and those aged 30-34 years (14.3 vs. 2.6%). However, gestational diabetes mellitus, premature birth, or low birth weight were not different between these two groups.

So, it seems that we should be concerned about hypertension in all pregnancies particularly in women underwent infertility treatments. 

### General considerations

BP measurement should be performed in all routine prenatal visits and more frequently in high risk patients. According to the last version of guideline of prenatal care, pregnant women should be visited every 4 weeks up to week 28, every 2 weeks thereafter until week 36 and then weekly up to time of delivery (41). If HDs were diagnosed, then BP should be checked weekly in milder forms and no association with preeclampsia, or twice and four times a week in moderate and severe forms, respectively (24).

Low salt diet is not advised, and consumption of calcium or magnesium supplements, fish oil derivatives, vitamins, antioxidants and garlic are also ineffective. No data supports using heparin and nitric oxide (18, 42). Weight reduction is not recommended. Moderate activity is suggested for patients with well-controlled chronic hypertension and it seems that there is no increase in incidence of preeclampsia in this group. If preeclampsia occurs, some physical activity restrictions may be required although it does not change maternal or fetal outcome (43). Bedtime low-dose aspirin (75-100 mg/day) should be started and continued until delivery to prevent preeclampsia although it has neutral effect on perinatal and maternal morbidity (17, 44). No evidence supports administration of dipyridamole (18).

### Drug therapy

In the first half of pregnancy, selected patients with pre- existing hypertension may need to discontinue their antihypertensive medications due to physiological drop in BP during this period, however, close monitoring is mandatory. New-onset hypertension during pregnancy is an indication for assessing proteinuria for early diagnosis of preeclampsia and should be repeated weekly. Fetal growth should be regularly monitored by ultrasonography (24). 

Most of the patients with pre-existing hypertension have mild-to-moderate increment in BP, thus physicians are not much worried about cardiovascular complications in this group. There are still scanty evidence about clinical benefits of administration of drugs to subjects with mild hypertension during pregnancy (Table 1) (45). Sometimes these patients should be hospitalized for at least a short period of time for confirmation of diagnosis and risk stratification specially for ruling out or ruling in preeclampsia for which the only effective treatment is termination of pregnancy.

Last European Society of Hypertension/ European Society of Cardiology (ESH/ESC) guidelines approved a systolic BP of 140 mmHg or a diastolic BP of 90 mmHg as the thresholds for antihypertensive treatment in pregnant women with one of the following criteria: gestational hypertension (with or without proteinuria), pre-existing hypertension superimposed by gestational hypertension, symptomatic hypertension and subclinical hypertension associated with end-organ damage.

The ESH/ESC thresholds are a systolic BP of 150 mmHg and a diastolic BP of 95 mmHg in any other conditions (Table 2) (43, 46).

Unfortunately, none of the antihypertensive agents could significantly reduce perinatal mortality (45). Continuation of current medication except for angiotensin- converting enzyme (ACE) inhibitors, ARBs, and direct renin inhibitors may be the best strategy in cases with pre-existing hypertension. Drug of choice is a-Methyldopa and labetalol shows comparable efficacy. Calcium channel blockers like nifedipine are drugs of second choice (17, 47). Dosages are stated in Table 3. Generally, diuretics should be avoided in all kinds of HDs as they may theoretically decrease placental blood flow (46); however, thiazides are mentioned as the second line therapy in some references (43, 48) as their teratogenicity or adverse effects have not been extensively studied (45). Mineralocorticoid receptor antagonists should never be prescribed (43). Nowadays, hydralazine is used only in hypertensive urgencies in intravenous form, although its chronic oral use showed no adverse effect. Prazosin and atenolol are no more recommended during pregnancy (17).

**Table 2:** Recommendations for the management of hypertension


Recommendations	Class of recommendation	Level of evidence

Non-pharmacological management for pregnant women with systolic BP of 140-150 mmHg or diastolic BP of 90-99 mmHg is recommended.	I	C
In women with gestational hypertension or pre-existing hypertension superimposed by gestational hypertension or with hypertension and subclinical organ damage or symptoms at any time during pregnancy, initiation of drug treatment is recommended at a BP of 140/90 mmHg. In any other circumstances, initiation of drug treatment is recommended if SBP ≥150 mmHg or DBP ≥95 mmHg.	I	C
Systolic BP ≥170 mmHg or diastolic BP ≥110 mmHg in a pregnant woman is an emergency, and hospitalization is recommended.	I	C
Induction of delivery is recommended in gestational hypertension with proteinuria with adverse conditions such as visual disturbances, coagulation abnormalities, or fetal distress	I	C
In preeclampsia associated with pulmonary edema, nitroglycerine given as an intravenous infusion, is recommended.	I	C
In severe hypertension, drug treatment with intravenous labetalol or oral methyldopa or nifedipine is recommended.	I	C
Women with pre-existing hypertension should be considered to continue their current medication except for ACE inhibitors, ARBs, and direct renin inhibitors under close BP-monitoring	IIa	C


From: ESC Guidelines on the management of cardiovascular diseases during pregnancy (46). BP; Blood pressure, SBP; Systolic blood pressure, DBP; Diastolic blood pressure, ACE; Angiotensin converting enzyme, and ARB; Angiotensin receptor blocker.

**Table 3:** Oral antihypertensive drugs commonly used in pregnancy


Drug	Indication	FDA category	Initial dose	Maximum dose	Potential side effects

Methyldopa	Often used as first line	B	125-250 mg BD	500 mg QID	Lethargy
Labetalol	Often used as first line	C	100 mg BD	200-400 mg QID	Exacerbation of asthma
Nifedipine (immediate release)	Second line or alternative first line	C	10-20 mg BD	40 mg BD	Concern for synergy with magnesium sulfate for neuromuscular depression


Modified from: Queensland Clinical Guideline: Hypertensive disorders of pregnancy (23) and Chronic Hypertension in Pregnancy (16). Category B; Animal reproduction studies have failed to demonstrate a risk to the fetus and there are no adequate and well-controlled studies in pregnant women, Category C; Animal reproduction studies have shown an adverse effect on the fetus and there are no adequate and well-controlled studies in humans, but potential benefits may warrant use of the drug in pregnant women despite potential risks, BD; Twice a day, and QID; Four times a day.

Severe hypertension is defined as BP ≥160/110 mmHg or systolic BP ≥170 mmHg and diastolic BP ≥110 mmHg. It is a medical emergency which requires hospital admission, rapid management and monitoring every 10 to 20 minutes depending on management strategy. In a systematic review of cases with very high BP, published from Cochrane library in 2013, 15 different antihypertensive medications in 35 trials were compared and the results showed that less persistent hypertension was seen with calcium channel blockers than with hydralazine and they also had less side effects compared to labetalol (50). The most popular plan is to start treatment with intravenous labetalol or oral nifedipine as soon as possible (Table 4) (49). Intravenous hydralazine is not the first choice according to some references (46) while there are references suggesting it as medication of first choice (48, 49). The optimal drug in hypertensive crises can be sodium nitroprusside. Intravenous magnesium sulfate is the preferable drug only for management of seizures and/or preventing eclampsia in selective cases of severe preeclampsia (46). If recurrent seizures occurred, alternative anticonvulsants like benzodiazepines, such as lorazepam and diazepam, phenytoin (Dilantin), and levetiracetam can be started (51). 

**Table 4:** Management of severe hypertension during pregnancy


Type of medication	Strategy

Hydralazine (IV)	5 mg IV bolus, then 10 mg every 20-30 minutes to a maximum of 25 mg, repeat in several hours as necessary
Labetalol (IV)	20 mg IV bolus over 2 minutes, then 40 mg 10 minutes later, 80 mg every 10 minutes for two additional doses to a maximum of 220 mg
Nifedipine (oral) (controversial)	10 mg po, repeat every 20 minutes to a maximum of 30 mg
Sodium nitroprusside (rarely used, usually when others fail)	0.25 μg/kg/minutes to a maximum of 5 μg/kg/minutes IV infusion Fetal cyanide poisoning may occur if used for more than 4 hours


Modified from: The Seventh Report of the Joint National Committee on Prevention, Detection, Evaluation, and Treatment of High Blood Pressure and Emergent therapy for acute-onset, severe hypertension during pregnancy and the postpartum period. Committee Opinion No. 623. American College of Obstetricians and Gynecologists (48, 49).

### Blood pressure goals in pregnancy

As a general agreement, diastolic BP should be kept above 80 mmHg (19). The American College of Obstetrics and Gynecology (ACOG) recommended to maintain BP between 120/80 and 160/105 mm Hg in pregnant women with HDs with no complications (52). Clinical practice guidelines of the Society of Obstetricians and Gynecologists of Canada emphasize that systolic BP of 130-155 mmHg and diastolic BP of 80-105 mmHg should be achieved in patients without any comorbidities and systolic BP of 130-139 mmHg and diastolic pressure of 80-89 mmHg are the goal BPs in cases with comorbidities like diabetes mellitus (53). According to a recent systematic review of patients with non -severe hypertension, target BP is variable and obviously dependent on the type of HDs in pregnancy and presence or absence of co- morbidities (1). So, if any form of end-organ dysfunction like left ventricular hypertrophy, chronic kidney disease and hypertensive retinopathy exists, the target BP would be 140/90 mmHg (5, 17, 20). In cases with no end-organ damages, target BP would be different. Accordingly in any form of HDs, the target is BP of 150/80-100 mmHg (5, 20), 130-159/80-105 mmHg (17), or, 160/110 mmHg, in patients with chronic hypertension, it would be120- 159/80-104 mmHg and at last in women with gestational hypertension or non-severe preeclampsia, the goal of BP is defined as 160/110 mmHg (54). 

### Time and mode of delivery

Uncontrolled severe hypertension is the most common maternal reason for preterm childbirth. There are no definite data for time of termination of pregnancy in patients with chronic hypertension specially when BP is well controlled. Delivery at term is suggested for cases with gestational hypertension. Patients with milder forms of preeclampsia can be observed up to gestational week 37 (17). Although the amount of proteinuria is not an important factor (43), severe preeclampsia and eclampsia necessitate urgent termination of pregnancy. Some cases with severe preeclampsia at low gestational age, can be closely observed for up to 34 weeks of pregnancy for better prognosis for child (23). In a recent study, adverse perinatal outcome of postponing delivery to later than 39 weeks of gestation (55) was shown; probably the best time for delivery in gestational hypertension is between weeks 38 and 39 (56).

Rout of delivery should be determined as obstetric indication, so vaginal delivery is the first choice (43). If vaginal delivery is planned, then active managing of the third stage of labor with oxytocin is recommended (17).

### Breast feeding

Although all antihypertensive medications are secreted in milk at very low levels, breast feeding is safe in hypertensive mothers. Lower dosages of captopril and enalapril are harmless. No adverse effect following administration of calcium channel blockers has been seen, although there are some controversies about nifedipine. Some references also recommend not to use amlodipine. Diuretics are best avoided as they potentially decrease milk production. Beta blockers other than atenolol can be used safely (16, 17, 23, 24, 43).

Methyldopa should not be used in this period due to some reports about the risk of post-natal depression (23), so must be discontinued within 2 days postpartum if has been prescribed during pregnancy (19, 24).

### Short and long-term cardiovascular complications

BP commonly rises immediately over the first 5 days after delivery (18.5% in a recent report) (57). Patients with HDs during pregnancy may become normotensive after labor but then becomes hypertensive in the first week. 

Women with a history of HDs during pregnancy, particularly when associated with early-onset pre-eclampsia, premature labor before 32 weeks of gestation, stillbirth, or IUGR are at high risk for developing cardiovascular complications such as hypertension, stroke and ischemic heart disease in later life. So, lifestyle modifications and close screening for sign and symptoms of cardiovascular diseases and also early detection of other risk factors are highly suggested in these groups. Notably, risk of subsequent hypertension or prehypertension in previously normotensive women with a history of HDs in pregnancy, is higher than others regardless of coincidence with gestational diabetes, even during the first year postpartum (58).

### Recurrence risk in a subsequent pregnancy

In a subsequent pregnancy, mothers who experienced hypertension in their first pregnancy are at greater risk specially if it was early onset or associated with HELLP syndrome. Although some other predictors like pre-pregnancy plasma volume have been suggested for risk assessment (59), they are not clinically relevant (60).

## Conclusion

HDs of pregnancy are highly prevalent, so do infertility treatments. It seems that elevated BP is somehow more common in women who received infertility treatments. Since hypertension is associated with many short and also long-term comorbidities and even perinatal mortality, this group of patients should be particularly screened, treated and followed up for high BP, although the hypertension treatment is not markedly different from those given to fertile mothers. 
